# A real-world 10-year follow-up analysis of survival and safety of CD20-targeted therapy in patients with Waldenstrom macroglobulinemia

**DOI:** 10.3389/fonc.2026.1733209

**Published:** 2026-03-02

**Authors:** Hengming Zhang, Xufei Huang, Yirong Zhu, Zhihong Zheng, Rong Zhan, Shaoyuan Wang

**Affiliations:** 1 Department of Pathology, WeiFang People's Hospital, Weifang, Shandong, China; 2 Fujian Institute of Hematology, Fujian Provincial Key Laboratory on Hematology, Hematology Department, Fujian Medical University Union Hospital, Fuzhou, Fujian, China

**Keywords:** CD20-targeted therapy, real-world study, safety, survival, Waldenstrom macroglobulinemia

## Abstract

**Objective:**

To evaluate the long-term survival and safety outcomes of CD20-targeted chemoimmunotherapy regimens in patients with Waldenstrom macroglobulinemia (WM) in a real-world single-center cohort from China.

**Methods:**

We conducted a retrospective analysis of 128 patients with WM who received CD20-based regimens at a single center from 2014 to 2024. Baseline characteristics, treatment efficacy, survival outcomes (OS and PFS) and safety profiles were compared among the four treatment groups.

**Results:**

Significant differences were found in age, disease burden, and bone marrow infiltration. Patients in the R-CHOP group had higher IgM levels and bone marrow infiltration rates. The FCR group achieved the longest OS (75.86 ± 22.05 months, *P* < 0.01). The RTX group showed the poorest outcomes, with a mortality rate of 75% within 12 months after relapse. ORR and consolidation therapy rates were similar across groups. The FCR group had the highest rate of grade ≥3 adverse events (60%), mainly leukopenia and thrombocytopenia. The R-CHOP group had a higher infection risk, while RTX was the safest.

**Conclusion:**

In this 10-year single-center real-world cohort from China, CD20-targeted chemoimmunotherapy achieved durable disease control with acceptable long-term safety in patients with WM. Differences in efficacy and toxicity profiles among FCR, BR, R-CHOP, and rituximab monotherapy support an individualized treatment approach that takes into account patient age, comorbidities, and tolerance. Because standardized quality-of-life instruments were not used in this retrospective study, our findings should be interpreted as reflecting survival and treatment-related toxicity rather than validated health-related quality of life.

## 1 Introduction

Waldenstrom Macroglobulinemia (WM) is a rare B-cell lymphoma, characterized by bone marrow lymphoplasmic cell infiltration and abnormal elevation of serum Immunoglobulin M (IgM) (1, 2). This disease predominantly affects the older population, with insidious onset and significant clinical heterogeneity. Common symptoms include anemia, bleeding tendency, hyperviscosity syndrome, peripheral neuropathy, and recurrent infection (3). Although traditional treatments, such as chemotherapy and immunomodulatory drugs, can partially alleviate symptoms and prolong survival, their efficacy is limited by cumulative toxicity, high recurrence rates, and the risk of secondary malignancies. Consequently, there is an urgent need for safer and more durable treatment strategies.

Given the indolent but incurable nature of WM, many patients will receive multiple lines of therapy over a prolonged disease course, and the cumulative burden of treatment-related toxicity is substantial. Most pivotal clinical trials were conducted in Western populations, and there is a paucity of real-world data from Asian cohorts regarding the long-term survival and safety of CD20-targeted chemoimmunotherapy (4–6). Moreover, access to novel agents such as Bruton tyrosine kinase (BTK) inhibitors has historically been limited in many regions of China (7–10). In this context, we conducted a 10-year single-center retrospective study to describe long-term survival, treatment patterns and toxicity of CD20-based regimens in Chinese patients with WM. Because standardized quality-of-life instruments were not available in this retrospective cohort, we focused on overall survival (OS), progression-free survival (PFS), treatment discontinuation and grade ≥3 adverse events as pragmatic clinical endpoints.

## 2 Materials and methods

### 2.1 Study flow chart

**Figure 1:**
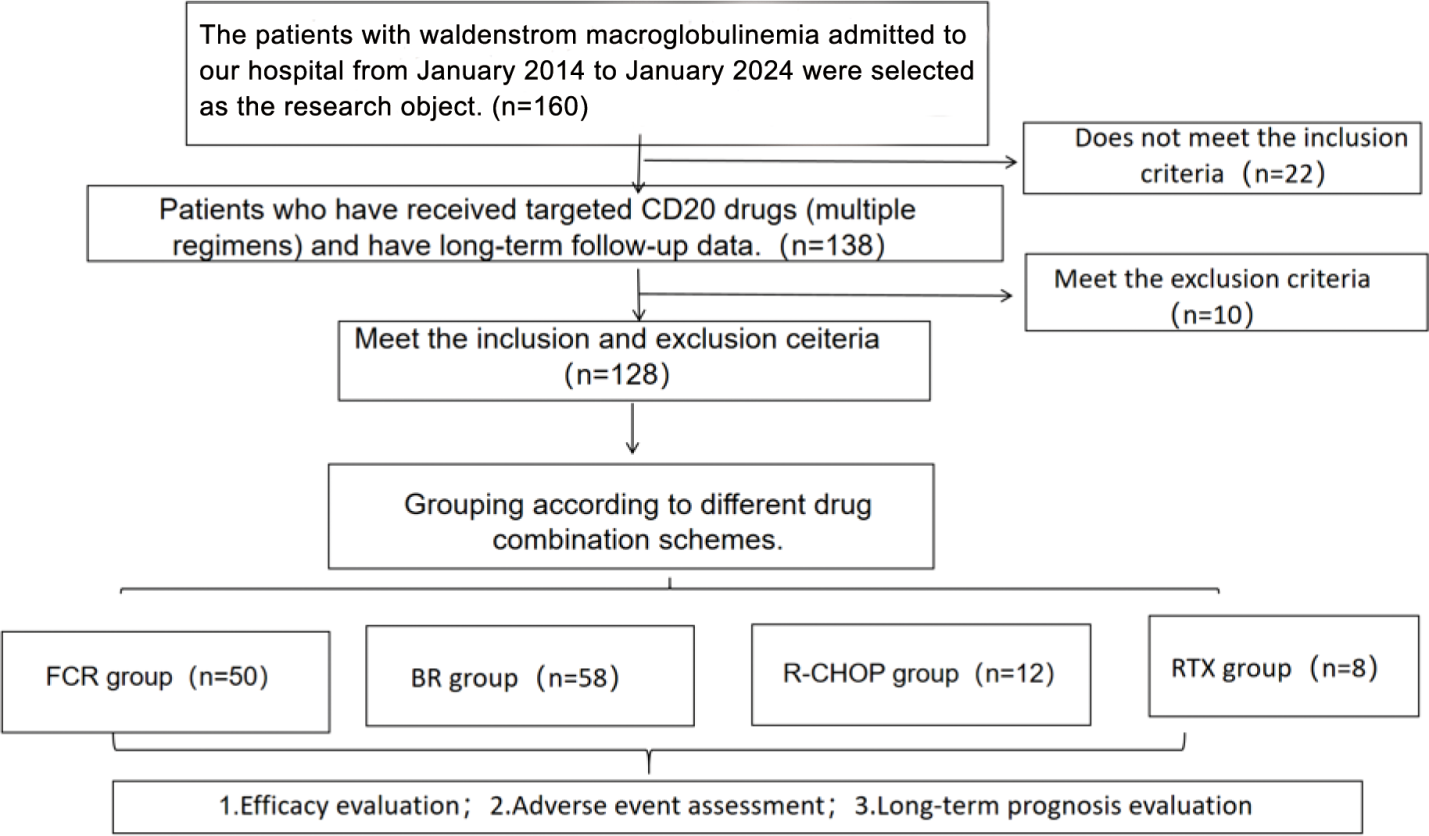
Study flow chart.

**Figure 1** shows the flow chart of this research.

### 2.2 Research object

From January 2014 to January 2024, we retrospectively identified 128 consecutive patients with Waldenström macroglobulinemia (WM) who were diagnosed and treated with CD20-based chemoimmunotherapy at Fujian Medical University Union Hospital, a tertiary referral center. The diagnosis of WM was established according to the World Health Organization (WHO) classification, based on bone marrow biopsy demonstrating lymphoplasmacytic lymphoma in association with any level of IgM monoclonal gammopathy. All bone marrow and, when available, lymph node biopsy specimens were reviewed by at least two experienced hematopathologists in the Department of Pathology at our institution; no additional external centralized pathology review was performed. All enrolled patients had clinically significant tumor burden at diagnosis, manifesting as immune-related cytopenia, hepatosplenomegaly, lymphadenopathy or other WM-related symptoms, and therefore met guideline-recommended indications for initiating chemoimmunotherapy. Eligible patients were required to be ≥18 years of age and to have received at least one cycle of a rituximab-based CD20-targeted regimen as initial treatment or first systemic therapy at our center. According to the different treatment regimens, patients were divided into four groups: fludarabine + cyclophosphamide + rituximab (FCR group, n = 50), bendamustine + rituximab (BR group, n = 58), rituximab + cyclophosphamide + doxorubicin + vincristine + prednisone (R-CHOP group, n = 12), and rituximab monotherapy (RTX group, n = 8). Patients who received their primary treatment outside our institution, had insufficient baseline or follow-up data, or were enrolled in interventional clinical trials were excluded. Baseline demographic and disease characteristics are summarized in **Table 1**, and detailed baseline laboratory parameters are provided in **Supplementary Table S1**. The study protocol was approved by the Ethics Committee of Fujian Medical University Union Hospital (approval number 2024KY070). Given the retrospective design and the use of anonymized data, the requirement for written informed consent was waived.

**Table 1:** Baseline demographic and disease characteristics of patients with Waldenström macroglobulinemia treated with CD20-based regimens.

Characteristic	FCR group (n=50)	BR group (n=58)	R-CHOP group (n=12)	RTX group (n=8)	χ²/F-value	P-value
Age (years, mean ± SD)	58.63 ± 10.24	65.37 ± 8.74	48.69 ± 6.72	75.44 ± 6.85	19.200	<0.001
Sex (male/female)	33/17	35/23	8/4	4/4	0.999	0.802
Age at WM diagnosis (years, mean ± SD)	57.26 ± 12.44	62.58 ± 13.31	47.86 ± 5.27	74.92 ± 8.15	9.654	<0.001
Follow-up duration (years, mean ± SD)	6.15 ± 2.14	5.93 ± 2.45	6.27 ± 2.24	5.81 ± 2.56	0.146	0.932
Enlarged spleen (yes, %)	12 (24.0)	30 (51.7)	12 (100.0)	8 (100.0)	33.497	<0.001
Enlarged lymph nodes (yes, %)	8 (16.0)	20 (34.5)	10 (83.3)	8 (100.0)	39.954	<0.001
MYD88 mutation (yes, %)	45 (90.0)	50 (86.2)	10 (83.3)	5 (62.5)	4.390	0.222
CXCR4 mutation (yes, %)	12 (24.0)	15 (25.9)	7 (58.3)	6 (75.0)	13.231	0.004
IPSSWM risk category					32.265	<0.001
Low	30	10	3	7		
Intermediate	18	35	6	1		
High	2	13	3	0		

Inclusion criteria: (1) Diagnosis of Waldenström Macroglobulinemia (WM) confirmed per the 2016 World Health Organization (WHO) criteria (11). Diagnosis requires integration of clinical manifestations, laboratory tests, and molecular genetic findings. Typical symptoms include chronic fatigue, weight loss, anemia, bleeding tendencies (such as skin ecchymosis), and peripheral neuropathy. Laboratory tests must demonstrate a significant increase in serum IgM levels (usually > 2g/dL), hematological abnormalities (anemia, leukopenia, thrombocytopenia), and lymphoplasmic infiltration confirmed by bone marrow biopsy (confirmed by immunohistochemistry or flow cytometry). Imaging (CT/PET-CT) may reveal splenomegaly or lymph node enlargement. Molecular genetic testing must identify the MYD88 L265P mutation (present in about 86% of patients), CXCR4 mutation (associated with drug resistance), and monoclonal IgM protein detection by immunoelectrophoresis. (2) Receiving at least one standard treatment regimen containing CD20 monoclonal antibody (rituximab) (including FCR, BR, R-CHOP, or monotherapy RTX) with completion of ≥3 treatment cycles; (3) Complete baseline data (including age, stage of IPSSWM, IgM level, proportion of bone marrow infiltration, etc.) and regular follow-up records (survival status, disease progression, adverse events, etc.) for ≥5 years; (4) Age ≥18 years, ability to comply with treatment and follow-up evaluations, and provision of informed consent. Case ascertainment & diagnostic criteria: For patients diagnosed before 2016, all source records were re−abstracted and uniformly reclassified according to the 2016 WHO criteria applied retrospectively. No patient failing 2016 criteria was retained.

Exclusion criteria: (1) History of other active solid tumors or hematological malignancies; (2) Severe cardiac, hepatic or renal insufficiency (e.g. LVEF<50%, ALT/AST>3 times the upper limit of normal, eGFR<30 mL/min/1.73m2) or uncontrollable infection; (3) Failure to complete the prescribed treatment regimen due to drug intolerance, financial constraints, or other reasons, or switching to non-CD20-targeted therapies (e.g., Bruton’s tyrosine kinase [BTK] inhibitors, bortezomib); (4) Pregnant or lactating women, or a documented history of grade ≥3 rituximab hypersensitivity (e.g., anaphylaxis, severe bronchospasm, or hypotension) despite premedication; non−severe infusion reactions alone were not exclusionary.

### 2.3 Treatment plan

Physicians chose among FCR, BR, R−CHOP, and rituximab monotherapy based on age, comorbidity burden, IPSSWM risk, tumor bulk (IgM level, marrow infiltration, lymphadenopathy), and frailty. Rituximab monotherapy was generally used in very elderly/frail patients. R−CHOP was reserved for suspected transformation or rapid cytoreduction needs (e.g., B symptoms, high LDH, high Ki−67, or high PET SUVmax), acknowledging that anthracycline−containing regimens are generally considered less preferable for typical WM and should be used cautiously. Management of high baseline IgM and rituximab−flare risk: For baseline IgM >4 g/dL or hyperviscosity, measures included therapeutic plasmapheresis within 24–48 hours when indicated; steroid pre−phase and/or cytoreductive chemotherapy prior to rituximab; deferral of rituximab until viscosity ≤4 cP or ≥50% IgM reduction; and enhanced monitoring during the first two infusions.

The treatment plan of this study strictly adhered to the National Comprehensive Cancer Network (NCCN) guidelines and the consensus of Chinese WM diagnosis and treatment experts (12). The specific medication plan of each group was as follows:

1. FCR group: Fludarabine 25 mg/m2 intravenous infusion (days 1-3) + cyclophosphamide 250 mg/m2 intravenous infusion (days 1-3) + rituximab 375 mg/m2 (day 0), administered every 28 days for a median of 6 cycles (range: 4–8).
2. BR group: Bendamustine 90 mg/m² (days 1-2) + rituximab 375 mg/m² (day 0), administered every 28 days for a median of 5 cycles (range: 4–6). For elderly patients (≥75 years), the bendamustine dose was reduced to 70 mg/m².
3. R-CHOP group: Rituximab 375 mg/m2 (day 0) + cyclophosphamide 750 mg/m2 (day 1) + doxorubicin 50 mg/m2 (day 1) + vincristine 1.4 mg/m2 (day 1, Max 2 mg) + prednisone 100 mg (days 1-5), administered every 21 days for a median of 6 cycles. All patients received G-CSF prophylactically.
4. Single-drug RTX group: Rituximab 375 mg/m2 was induced once a week ×4 weeks, and then maintained once every 3 months ×2 years (a total of 8 doses). Antiallergic pretreatment (promethazine + dexamethasone) was routinely administered across all regimens.

All CD20-targeted regimens in this cohort used rituximab as the anti-CD20 monoclonal antibody component; no patients received obinutuzumab or other second-generation anti-CD20 antibodies during the study period.

### 2.4 Data collection

Baseline clinical features were collected using the electronic medical record system, including age, international prognostic scoring system for WM (IPSSWM), immunoglobulin M (IgM) levels, bone marrow infiltration percentage, and gene mutation status. Treatment response data (overall response rate [ORR], complete response rate [CRR]) and long-term follow-up information (overall survival [OS], progression-free survival [PFS], and relapse-related mortality) were also recorded.

1. IPSSWM scoring criteria (13): based on five clinical factors: age ≥ 65 years, hemoglobin ≤ 115 g/L, platelet count ≤ 100 ×10^9^/L, β_2_-microglobulin > 3 mg/L, and serum IgM ≥ 70 g/L. Each factor is assigned 1 point. Patients were then categorized into low-risk (score = 0 or 1 with age < 65), intermediate-risk (score = 2 or age ≥ 65), and high-risk (score > 2) groups. Higher scores were associated with shorter median overall survival and lower 5-year survival rates.
2. Response Evaluation Criteria (14): Treatment efficacy was assessed according to the latest Chinese Guidelines for the Diagnosis and Treatment of Lymphoplasmacytic Lymphoma/Waldenström’s Macroglobulinemia (2022 Edition) and NCCN Clinical Practice Guidelines. Response categories included complete response (CR), very good partial response (VGPR), partial response (PR), minimal response (MR), stable disease (SD), and progressive disease (PD), primarily based on changes in serum IgM levels and clinical symptoms. Specifically, CR was defined as the disappearance of serum monoclonal IgM protein, resolution of lymphadenopathy and splenomegaly, and no disease-related symptoms. PD was defined as an increase in serum IgM levels of ≥25% from the lowest point (nadir), or the appearance of new/worsening clinical manifestations such as anemia, thrombocytopenia, or lymphadenopathy. Patients who achieved CR or VGPR were evaluated for consolidation therapy (e.g., additional rituximab cycles), depending on clinical status and comorbidities.
3. Progression-free survival (PFS) was the time from treatment initiation to Progression/death, and Overall survival (OS) was the time from diagnosis to death, both of which were the core efficacy endpoint. Transformation to aggressive lymphoma (e.g., diffuse large B-cell lymphoma) was defined as the development of a biopsy-proven high-grade B-cell lymphoma during follow-up after an initial diagnosis of WM.
4. Common terminology criteria for adverse events 4.0 (CTCAE 4.0) (15): Adverse events were graded on a 5-level scale: Grade 1 (mild, no intervention required), Grade 2 (moderate, affecting activity but not requiring hospitalization), Grade 3 (severe, requiring hospitalization), Grade 4 (life-threatening), and Grade 5 (death). Common grade ≥3 events include myelosuppression (leukopenia, thrombocytopenia, anemia), infection, and gastrointestinal reactions. All events were recorded per CTCAE standards to assess treatment safety.

### 2.5 Statistical analysis

Overall survival (OS) was defined as the time from diagnosis of WM to death from any cause or last follow-up, and progression-free survival (PFS) was defined as the time from initiation of the index CD20-based regimen to documented disease progression, relapse, or death, whichever occurred first. Patients who were alive without documented progression at the time of last contact were censored at that date. Patients who were lost to follow-up were censored at the date of last documented clinical assessment. OS and PFS were estimated using the Kaplan–Meier method, and survival curves were compared between treatment groups using the log-rank test. Continuous variables were compared using the t test or Wilcoxon rank-sum test, as appropriate, and categorical variables were compared using the chi-square or Fisher’s exact test.

We initially considered fitting multivariable Cox proportional hazards models to identify independent predictors of OS and PFS; however, the number of death and progression events was limited, particularly in the smaller treatment groups, and several key baseline covariates were missing in a non-negligible proportion of patients. In view of the high risk of overfitting and unstable estimates under these conditions, we chose to present descriptive Kaplan–Meier curves and univariable log-rank p values as our primary survival analyses.

## 3 Result

### 3.1 Baseline data

A total of 128 patients with WM were included in the study, and the median follow-up duration was 6.20 ± 2.33 years. At baseline, the four treatment groups differed significantly with respect to age at diagnosis, splenomegaly, lymphadenopathy, bone marrow infiltration and several other clinical characteristics (all P < 0.05). In terms of disease burden, patients in the R-CHOP group had the highest median serum IgM level (12.04 g/dL) and the greatest proportion of bone marrow involvement (45.18%), consistent with more aggressive disease in this cohort. The distribution of IPSSWM risk categories also varied across the four groups, with a higher proportion of high-risk patients observed in the BR and R-CHOP groups, which likely influenced regimen selection in routine clinical practice.

With respect to molecular characteristics, MYD88 mutations were detected in 110 of 128 patients (85.9%), with similar proportions across the four treatment groups, whereas CXCR4 mutations were present in 40 patients (31.3%), with a numerically higher frequency in the BR and R-CHOP groups. MYD88 wild-type and CXCR4 wild-type cases, including a small number of patients with a double wild-type genotype, were also included in this cohort; however, their numbers were too limited to permit separate subgroup analyses. In exploratory analyses, no clear or consistent association was observed between MYD88 or CXCR4 mutation status and treatment response or survival, although the study was not powered to detect modest genotype-associated differences in outcomes.

These imbalances in baseline demographic, clinical and molecular characteristics were not adjusted for by multivariable methods and may therefore affect subsequent efficacy comparisons. Key baseline demographic and disease characteristics are summarized in **Table 1**, and more detailed baseline laboratory parameters are presented in **Supplementary Table S1**.

### 3.2 Comparison of clinical efficacy among four groups

**Figure 2:**
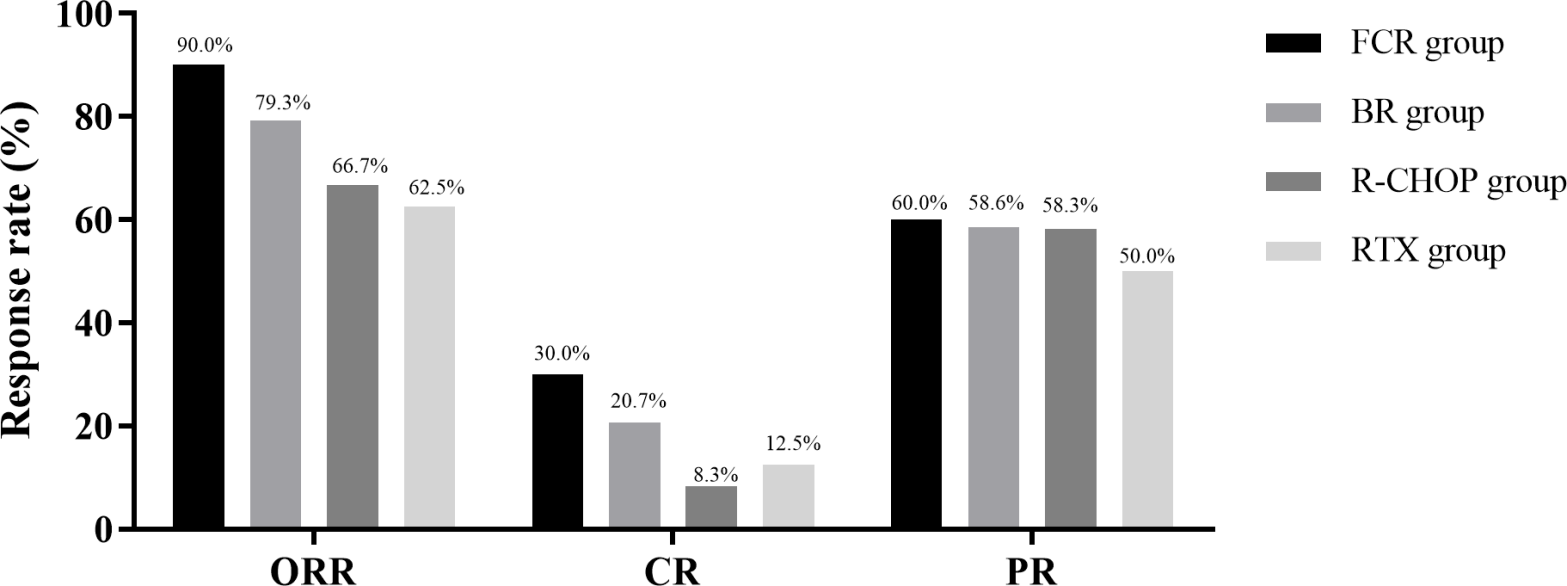
Comparison of treatment efficacy among the four groups. The x-axis shows the four treatment groups (FCR, BR, R-CHOP and rituximab monotherapy), and the y-axis shows the proportion of patients achieving at least a partial response (%).

The FCR group achieved the highest treatment response among the four regimens. The overall objective response rate (ORR) in the FCR group was 90.0% (45/50), compared with 79.3% (46/58) in the BR group, 66.7% (8/12) in the R-CHOP group, and 62.5% (5/8) in the RTX group. The complete response (CR) rate was also highest in the FCR group at 30.0% (15/50), whereas the CR rates in the BR, R-CHOP and RTX groups were 20.7% (12/58), 8.3% (1/12), and 12.5% (1/8), respectively. The partial response (PR) rates were 60.0% (30/50) in the FCR group, 58.6% (34/58) in the BR group, 58.3% (7/12) in the R-CHOP group, and 50.0% (4/8) in the RTX group (**Figure 2**).

**Figure 3:**
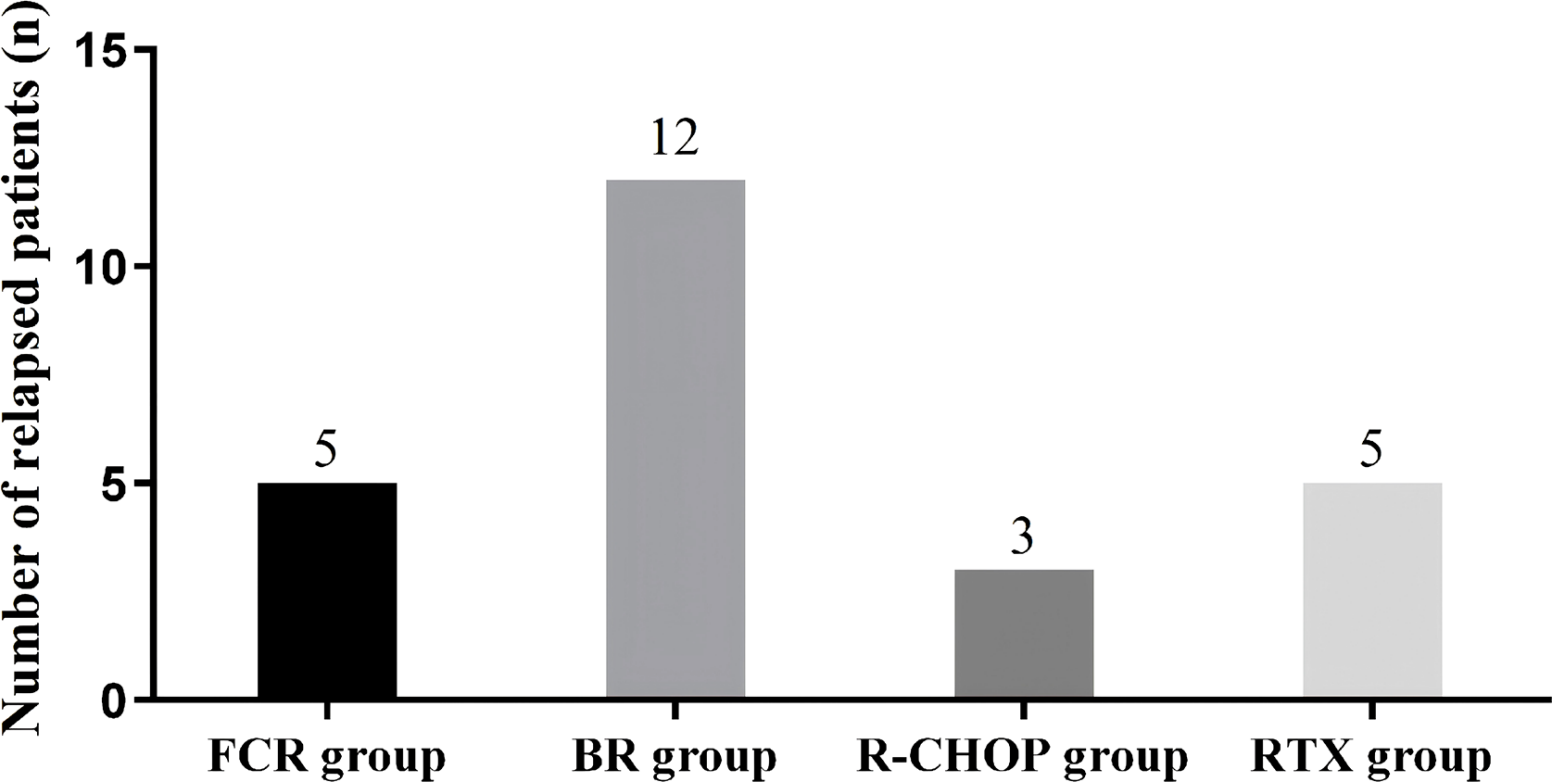
Comparison of relapse events among the four treatment groups. The x-axis shows the four regimens (FCR, BR, R-CHOP and rituximab monotherapy), and the y-axis shows the number of patients who experienced disease relapse during follow-up.

Although the overall ORR did not differ significantly among the four groups, the FCR regimen yielded the highest depth of response as reflected by the superior CR rate. The BR and R-CHOP regimens showed moderate efficacy in patients with higher-risk disease. However, the BR group exhibited a relatively higher recurrence rate, while the R-CHOP group was associated with a markedly higher toxicity burden, which may limit its clinical applicability despite its cytoreductive potential (**Figure 3**).

**Figure 4:**
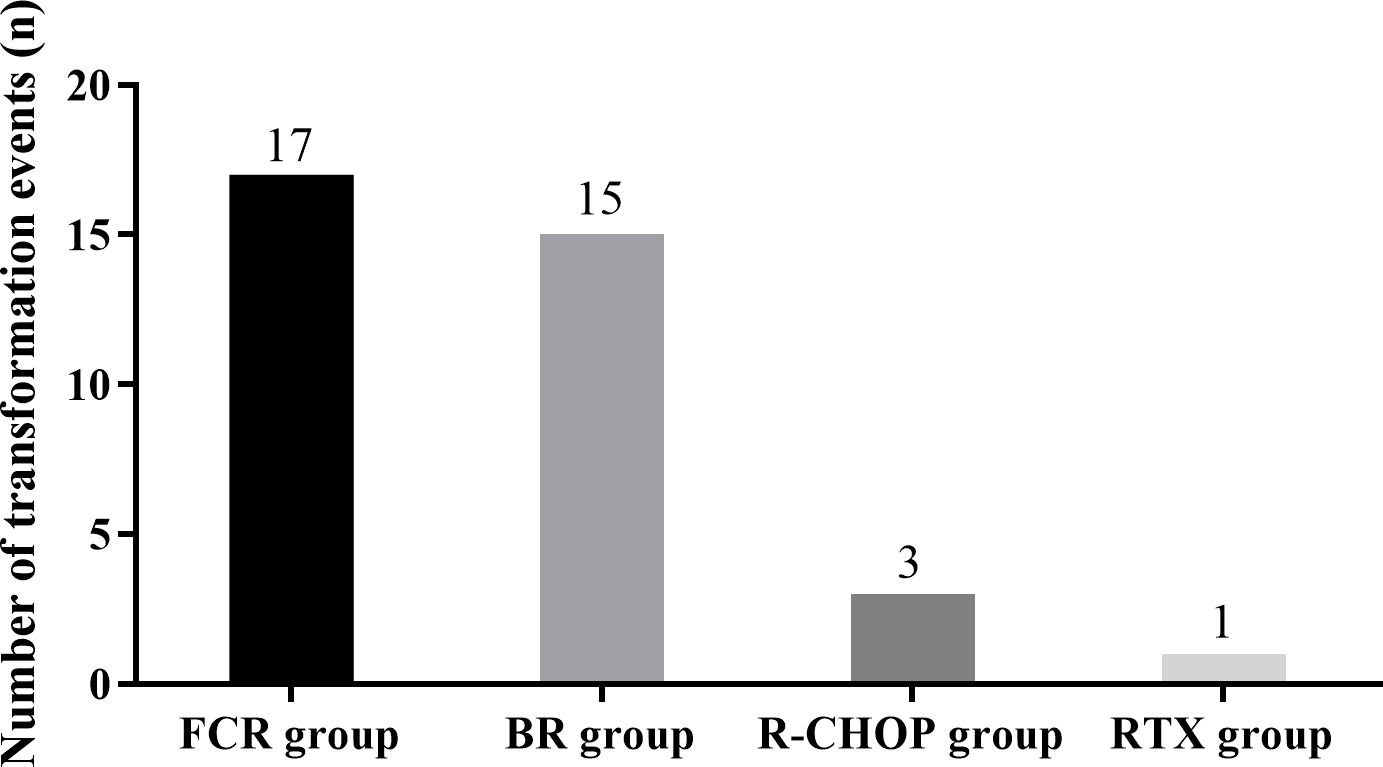
Comparison of consolidation therapy across the four treatment groups. The x-axis shows the four regimens (FCR, BR, R-CHOP and rituximab monotherapy), and the y-axis shows the number of patients who underwent consolidation treatment.

The proportion of patients receiving consolidation therapy remained low across all treatment groups, and no statistically significant difference was observed among the four groups (P = 0.567, **Figure 4**).

### 3.3 Survival assessment

**Figure 5:**
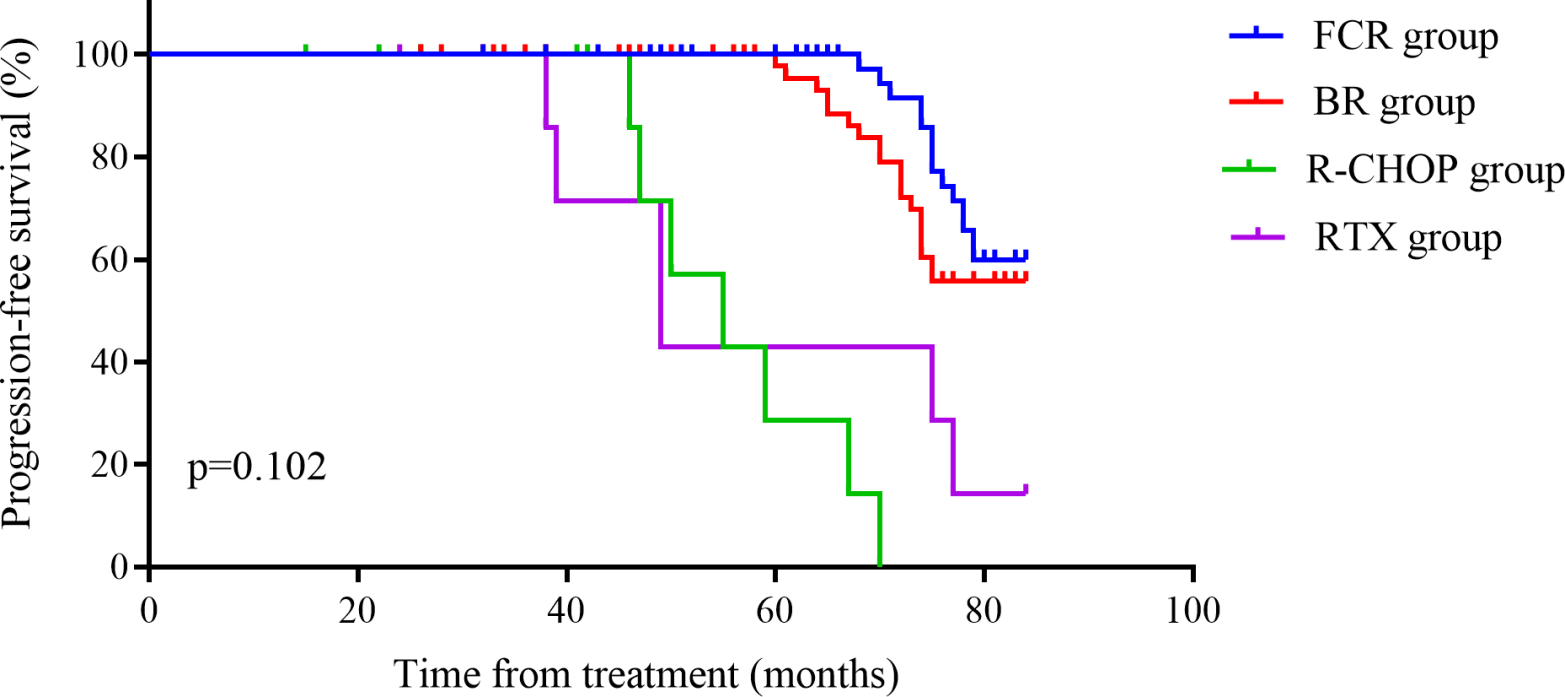
Kaplan–Meier curves of progression-free survival in the four treatment groups. The x-axis represents time from initiation of CD20-based therapy (months), and the y-axis represents the estimated probability of remaining progression-free. Separate curves are shown for FCR, BR, R-CHOP and rituximab monotherapy.

**Figure 6:**
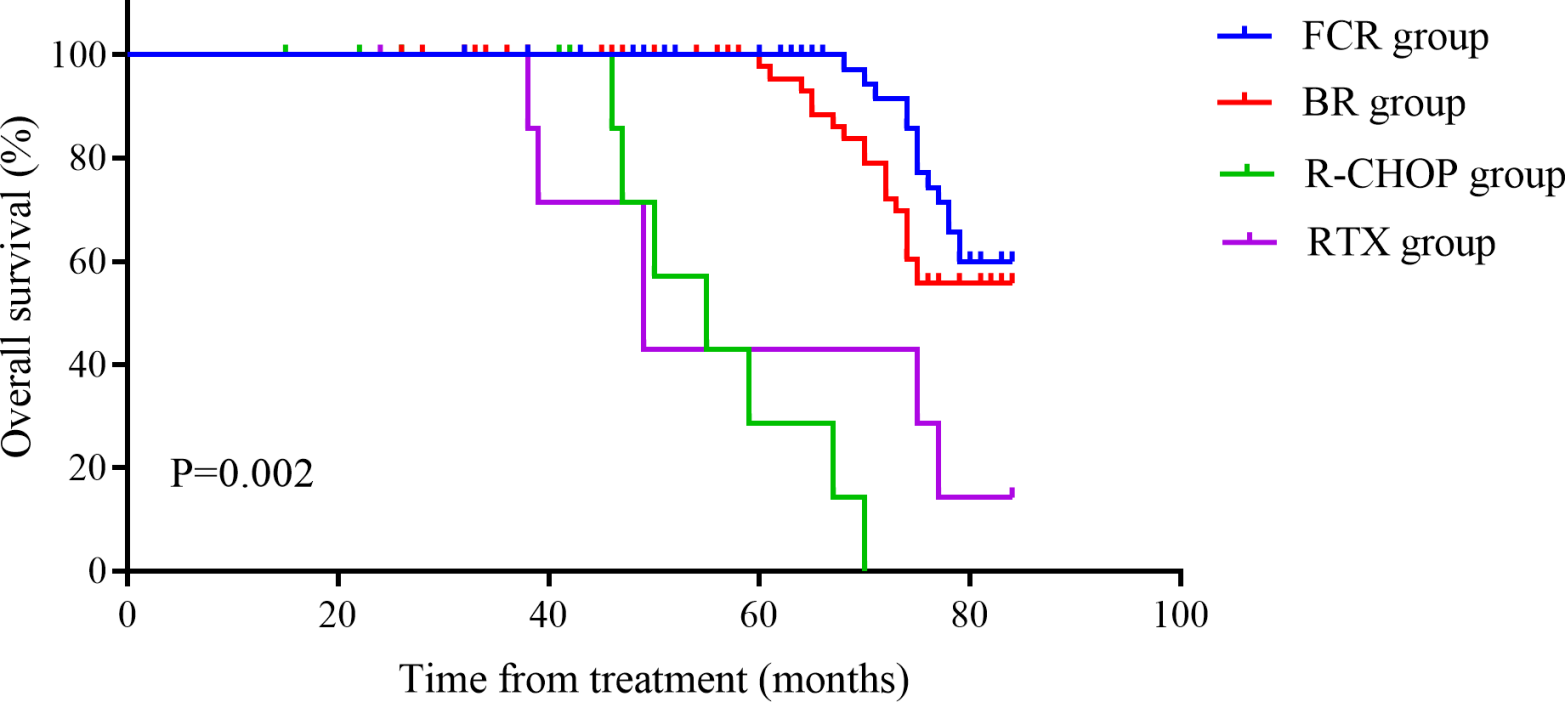
Overall survival (OS) Kaplan-Meier curve of the four groups. The x-axis represents time from diagnosis (months), and the y-axis represents the estimated probability of overall survival. Separate curves are shown for FCR, BR, R-CHOP and rituximab monotherapy.

The OS of FCR group was (75.86 ± 22.05) months, which was significantly better compared to other groups (P = 0.002). Although the PFS was 39.3 months, the difference was not statistically significant (P = 0.102), possibly due to an insufficient sample size. See **Figures 5**, **6** for details. Histologic transformation to an aggressive lymphoma was rarely observed in this cohort, and the small number of transformation events precluded meaningful comparative analysis between treatment groups.

### 3.4 Safety analysis

Significant differences in treatment safety were observed among the four groups, with the highest incidence of grade ≥3 adverse events was reported in the FCR group (60%), mainly due to bone marrow suppression (50%), including leukopenia (46%) and thrombocytopenia (40%). Bone marrow suppression and infection risk (41.7%) were more prominent in the R-CHOP group, likely due to the intensity of chemotherapy. In contrast, the RTX group demonstrated the best safety profile, with a grade ≥3 adverse event rate of 25%, though its efficacy was limited, suggesting suitability for elderly patients or those with poor tolerance. Combination chemotherapy groups showed higher rates of infection and gastrointestinal symptoms, necessitating close clinical monitoring. The distribution of grade ≥3 adverse events across the four regimens is summarized in **Table 2**, and additional laboratory safety data are presented in **Supplementary Table S1**.

**Table 2:** Types and proportion of grade 3 or higher adverse events during treatment.

Characteristic	FCR treatment group (n=50)	BR treatment group (n=58)	R-CHOP treatment group (n=12)	Single drug RTX group (n=8)	*χ^2^/F*-value	P-value
Overall ≥ Grade 3 adverse event rate (≥3 AE Rate, %)	30 (60.00)	23 (39.66)	10 (83.33)	2 (25.00)	11.788	0.008
Myelosuppression (Bone Marrow Suppression, %)	25 (50.00)	14 (24.14)	9 (75.00)	1 (12.50)	16.915	0.001
Leukopenia (Leukopenia, %)	23 (46.00)	12 (20.69)	8 (66.67)	1 (12.50)	15.055	0.002
Thrombopenia (Thrombocytopenia, %)	20 (40.00)	9 (15.52)	7 (58.33)	1 (12.50)	14.158	0.003
Anaemia (Anemia, %)	12 (24.00)	6 (10.34)	6 (50.00)	0 (0.00)	13.133	0.004
Gastrointestinal symptoms (Gastrointestinal, %)	7 (14.00)	3 (5.17)	5 (41.67)	0 (0.00)	14.119	0.003
Be infected with (Infection, %)	7 (14.00)	3 (5.17)	5 (41.67)	0 (0.00)	14.119	0.003
Intravenous infusion reaction (Infusion Reaction, %)	2 (4.00)	3 (5.17)	2 (16.67)	0 (0.00)	3.592	0.309
Anaphylaxis (Allergic Reactions, %)	2 (4.00)	3 (5.17)	2 (16.67)	0 (0.00)	3.592	0.309
Other adverse events (Other AEs, %)	7 (14.00)	3 (5.17)	2 (16.67)	1 (12.50)	2.994	0.393

## 4 Discussion

According to the International Lymphoma Research Organization (16), the annual incidence of WM is approximately 3 to 5 cases per million people, increasing gradually with age (median age at diagnosis: ~68 years). The exact etiology remains unclear, but studies (17) suggest that genetic predisposition, environmental factors, and abnormal regulation of the immune system may interact in the pathogenesis of WM. The clinical manifestations of WM range from asymptomatic to severe anemia, bleeding tendencies, neurological symptoms, and organ dysfunction. Because of its rarity, the diagnosis and treatment of WM is often challenging, requiring integration of clinical presentation, laboratory findings, and molecular/genetic characteristics. CD20-targeted therapy, an immunotherapy directed against B-cell surface antigens, offers promising prospects for WM patients. CD20, exclusively expressed on B cells, enables therapies to selectively target and eliminate CD20-positive tumor cells, inhibiting their growth and spread. In WM treatment, targeted CD20 therapy effectively relieve symptoms and may improve the long-term quality of life of patients. However, the long-term effects of treatment and side effects management still need in-depth research to ensure that patients can get the best treatment results and quality of life.

This study revealed significant heterogeneity in age, disease burden (IgM level, proportion of bone marrow infiltration), and IPSSWM score among the four groups (P<0.05). Notably, patients in the R-CHOP group exhibited higher IgM levels and bone marrow infiltration, with a greater proportion classified as moderate-to-high risk. This trend likely reflects clinical practice, where aggressive chemotherapy regimens are preferred for patients with high tumor burden, aligning with NCCN guidelines recommending anthracycline-containing regimens for medium-to-high-risk patients. However, baseline imbalances can have confounding effects on inter-group comparisons of OS and PFS. For instance, the FCR group included younger patients with a higher proportion of IPSSWM low-risk cases, suggesting that their therapeutic advantage may partly stem from favorable baseline characteristics. In terms of efficacy, the FCR group demonstrated the highest OS and CR rates, confirming the core role of fludarabine combined with CD20 monoclonal antibody in low-risk WM. This efficacy likely results from fludarabine’s deep clearance of lymphoplasmacytic cells, synergizing with rituximab’s ADCC effect. However, the FCR group experienced a high incidence of grade ≥3 myelosuppression (60%, including 46% leukopenia), significantly exceeding other groups (P = 0.008). This finding aligns with prior reports of fludarabine-associated long-term hematopoietic injury (18, 19). Although PFS was not statistically different in the FCR group, OS was significantly prolonged, which may suggest that the regimen indirectly improves survival by delaying disease progression or has a positive effect on treatment sensitivity after relapse. However, long-term myelosuppression may increase the risk of infection and secondary malignancy, and the benefit-risk ratio needs to be carefully balanced in young, low-risk patients.

In addition, the BR group accounted for the highest proportion of intermediate-high-risk patients in this study, with its OS and safety profile (≥ grade 3 AE 39.66%) showed its applicability in high-risk populations. The alkylating properties of bendamustine can effectively penetrate the bone marrow microenvironment and synergize with rituximab to enhance tumor cell killing. However, the higher recurrence rate in this group may be related to the limited efficacy of bendamustine in patients with CXCR4 mutations. Recent studies (20) suggest that BTK inhibitors combined with CD20 mab can overcome CXCR4-mediated drug resistance, suggesting potential for sequential targeted therapies with BR in future research. Although the R-CHOP group achieved disease control in patients with extremely high tumor burden, the incidence of grade ≥3 AE was as high as 83.33%, which significantly limited its clinical application. This toxicity is largely attributable to the myelosuppressive and immunosuppressive effects of anthracyclines (e.g., doxorubicin). Notably, this group had the youngest patients, indicating a clinical preference for intensive chemotherapy in younger, high-risk individuals. However, the elevated toxicity increases the risk of treatment discontinuation. Recent studies have advocated replacing R-CHOP with non-anthracycline regimens (e.g. BR) to reduce toxicity, and these results further support this trend.

Transformation of WM into an aggressive lymphoma, most commonly diffuse large B-cell lymphoma, represents a clinically meaningful but relatively infrequent event that is often underreported in routine practice. In our cohort, histologic transformation was rare, and the small number of events did not allow for robust analysis of risk factors or potential associations with specific treatment regimens. Nevertheless, transformation remains an important long-term concern for patients with indolent B-cell malignancies, and recent work by Maher et al. has highlighted key molecular mechanisms underlying the evolution from indolent to aggressive B-cell neoplasms and potential therapeutic implications (21). Future prospective studies with systematic long-term follow-up and standardized reporting of transformation events are needed to better define the incidence, predictors and outcomes of WM transformation in different treatment eras.

The therapeutic landscape of WM has evolved substantially over the past decade. Recent NCCN (Version 2.2024) and ESMO updates now recommend Bruton tyrosine kinase (BTK) inhibitors, such as zanubrutinib or ibrutinib with or without rituximab, as preferred options for many patients with symptomatic WM, particularly in the frontline and relapsed/refractory settings (17, 22). These agents have demonstrated high response rates and durable disease control with a generally favorable toxicity profile compared with traditional chemoimmunotherapy (17, 23–25). However, access to BTK inhibitors has been gradually implemented and remains variable across different regions in China, and long-term, real-world data from Asian populations are still relatively sparse. Our single-center cohort, largely treated with FCR, BR, R-CHOP and rituximab monotherapy, therefore provides an important benchmark for survival and safety outcomes in patients who either received treatment before BTK inhibitors became widely available or were not candidates for continuous BTK inhibition. In the current era, our findings support the notion that chemoimmunotherapy remains a reasonable option for selected patients with good performance status, while BTK inhibitor–based regimens should be considered for those with higher comorbidity burden or who are less tolerant of cytotoxic chemotherapy.

Finally, we did not prospectively collect patient-reported outcomes or administer validated quality-of-life instruments (such as EORTC QLQ-C30 or FACT-Lym), and therefore our findings should not be interpreted as demonstrating long-term health-related quality-of-life benefits but rather as describing survival and long-term safety in routine clinical practice. In addition, because the number of events was limited and some baseline covariates were incompletely captured in this retrospective cohort, we did not pursue formal multivariable Cox regression analyses, and the absence of hazard ratios and 95% confidence intervals should be considered when interpreting the survival comparisons among treatment groups. Moreover, although most patients had MYD88-mutated disease and approximately one-third harbored CXCR4 mutations, the number of MYD88 wild-type and CXCR4 wild-type (including double wild-type) cases was small and molecular data were incomplete for some patients, so we were unable to perform robust genotype-stratified survival analyses.

## 5 Conclusion

In summary, the management of WM needs to be individualized to balance long-term efficacy and safety. In our 10-year single-center real-world cohort, CD20-based chemoimmunotherapy provided durable disease control with acceptable toxicity. The FCR regimen appears most suitable for younger patients with low-risk disease and good performance status, whereas BR can be considered an effective first-line option for many patients with intermediate risk. Rituximab monotherapy may be appropriate for elderly or frail patients who are unable to tolerate intensive chemoimmunotherapy, while R-CHOP remains reserved for selected patients with higher tumor burden or aggressive clinical features. In the current era, when BTK inhibitor–based regimens are increasingly available, our results provide a useful benchmark for survival and safety outcomes in patients who receive or continue to receive CD20-based chemoimmunotherapy, particularly in settings where novel agents are inaccessible or contraindicated. Key limitations of this study include its single-center retrospective design, potential selection bias, small sample sizes in the R-CHOP (n = 12) and rituximab monotherapy (n = 8) groups, and inter-group heterogeneity in baseline characteristics such as IPSSWM score and CXCR4 mutation status. The median follow-up of 6.1 years and incomplete capture of very late events also limit our ability to fully assess secondary malignancies and other long-term complications. Furthermore, we did not prospectively collect patient-reported outcomes or administer validated quality-of-life instruments, so our findings should be interpreted as reflecting survival and treatment-related toxicity rather than formal health-related quality of life. Future multicenter prospective studies with systematic molecular profiling and incorporation of BTK inhibitor–based strategies, as well as standardized patient-reported outcomes, are needed to refine risk-adapted treatment algorithms and optimize long-term care for patients with WM.

## Data Availability

The raw data supporting the conclusions of this article will be made available by the authors, without undue reservation.

## References

[1] Grunenberg A Buske C . How to manage waldenstrom’s macroglobulinemia in 2024. Cancer Treat Rev. (2024) 125:102715. doi: 10.1016/j.ctrv.2024.102715, PMID: 38471356

[2] Gertz MA . Waldenstrom macroglobulinemia: 2023 update on diagnosis, risk stratification, and management. Am J Hematol. (2023) 98:348–58. doi: 10.1002/ajh.26796, PMID: 36588395 PMC10249724

[3] Kapoor P Ansell SM Fonseca R Chanan-Khan A Kyle RA Kumar SK . Diagnosis and management of waldenstrom macroglobulinemia: mayo stratification of macroglobulinemia and risk-adapted therapy (mSMART) guidelines 2016. JAMA Oncol. (2017) 3:1257–65. doi: 10.1001/jamaoncol.2016.5763, PMID: 28056114 PMC5556979

[4] Fotiou D Theodorakakou F Kastritis E . Monoclonal antibody-based therapies for Waldenstrom’s macroglobulinemia. Leuk Res Rep. (2022) 17:100324. doi: 10.1016/j.lrr.2022.100324, PMID: 35572915 PMC9098391

[5] Sermer D Sarosiek S Branagan AR Treon SP Castillo JJ . SOHO State of the Art Updates and Next Questions: Targeted therapies and emerging novel treatment approaches for Waldenstrom Macroglobulinemia. Clin Lymphoma Myeloma Leuk. (2022) 22:547–56. doi: 10.1016/j.clml.2022.02.005, PMID: 35339405

[6] Carlson AK Amin M Cohen JA . Drugs targeting CD20 in multiple sclerosis: pharmacology, efficacy, safety, and tolerability. Drugs. (2024) 84:285–304. doi: 10.1007/s40265-024-02011-w, PMID: 38480630 PMC10982103

[7] Adam Z Pour L Zeman D Krejci M Boichuk I Krejci M . Waldenstrom’s macroglobulinemia - clinical symptoms and review of therapy yesterday, today and tomorrow. Klin Onkol. (2023) 36:177–91. doi: 10.48095/ccko2023177, PMID: 37353346

[8] Khan AM . The use of bruton tyrosine kinase inhibitors in waldenstrom’s macroglobulinemia. J Pers Med. (2022) 12:676. doi: 10.3390/jpm12050676, PMID: 35629099 PMC9146645

[9] Takarada A Momose H Kurita N Matsuoka R Nakamura N Sakamoto T . Lymphoplasmacytic lymphoma accompanied by severe myelofibrosis. Rinsho Ketsueki. (2023) 64:54–9. doi: 10.11406/rinketsu.64.54, PMID: 36775308

[10] Chmielewska N Szyndler J . Targeting CD20 in multiple sclerosis - review of current treatment strategies. Neurol Neurochir Pol. (2023) 57:235–42. doi: 10.5603/PJNNS.a2023.0022, PMID: 36999373

[11] Ravi G Kapoor P . Current approach to waldenstrom macroglobulinemia. Cancer Treat Res Commun. (2022) 31:100527. doi: 10.1016/j.ctarc.2022.100527, PMID: 35149375

[12] Zinzani PL Mauro FR Tedeschi A Varettoni M Zaja F Barosi G . Unmet clinical needs in the use of zanubrutinib in Malignant lymphomas (Waldenstrom macroglobulinemia, marginal zone lymphoma and mantle cell lymphoma): A consensus-based position paper from an *ad hoc* expert panel. Hematol Oncol. (2023) 41:795–808. doi: 10.1002/hon.3172, PMID: 37165730

[13] Morel P Duhamel A Gobbi P Dimopoulos MA Dhodapkar MV McCoy J . International prognostic scoring system for Waldenstrom macroglobulinemia. Blood. (2009) 113:4163–70. doi: 10.1182/blood-2008-08-174961, PMID: 19196866

[14] A. Hematology Oncology Committee of China Anti-Cancer C.M.A. Chinese Society of Hematology and M. Chinese Working Group of Walderstrom . Chinese guideline for diagnosis and treatment of lymphoplasmacytic lymphoma/Walderstrom macroglobulinemia (2022). Zhonghua Xue Ye Xue Za Zhi. (2022) 43:624–30. doi: 10.3760/cma.j.issn.0253-2727.2022.08.002, PMID: 36709145 PMC9593020

[15] Chen AP Setser A Anadkat MJ Cotliar J Olsen EA Garden BC . Grading dermatologic adverse events of cancer treatments: the Common Terminology Criteria for Adverse Events Version 4.0. J Am Acad Dermatol. (2012) 67:1025–39. doi: 10.1016/j.jaad.2012.02.010, PMID: 22502948

[16] McMaster ML . The epidemiology of Waldenstrom macroglobulinemia. Semin Hematol. (2023) 60:65–72. doi: 10.1053/j.seminhematol.2023.03.008, PMID: 37099032 PMC10351685

[17] Kumar SK Callander NS Adekola K Anderson LD Jr. Baljevic M Baz R . Waldenstrom macroglobulinemia/lymphoplasmacytic lymphoma, version 2.2024, NCCN clinical practice guidelines in oncology. J Natl Compr Canc Netw. (2024) 22:e240001. doi: 10.6004/jnccn.2024.0001, PMID: 38244272

[18] Lin F Zhang Y Han T Cheng Y Mo X Wang J . A modified conditioning regimen based on low-dose cyclophosphamide and fludarabine for haploidentical hematopoietic stem cell transplant in severe aplastic anemia patients at risk of severe cardiotoxicity. Clin Transplant. (2022) 36:e14514. doi: 10.1111/ctr.14514, PMID: 34655493

[19] Vergara-Cadavid J Johnson PC Kim HT Yi A Sise ME Leaf DE . Clinical features of acute kidney injury in the early post-transplantation period following reduced-intensity conditioning allogeneic hematopoietic stem cell transplantation. Transplant Cell Ther. (2023) 29:455 e1–9. doi: 10.1016/j.jtct.2023.03.029, PMID: 37015320 PMC10330095

[20] Feng L Gao X Jiao Z Wang Z Min F . BTK inhibitor combined with anti-PD-1 monoclonal antibody for the treatment of CD20-negative primary central nervous system lymphoma: A case report. Oncol Lett. (2023) 25:48. doi: 10.3892/ol.2022.13634, PMID: 36644138 PMC9811622

[21] Maher N Mouhssine S Matti BF Alwan AF Gaidano G . Molecular mechanisms in the transformation from indolent to aggressive B cell Malignancies. Cancers (Basel). (2025) 17:907. doi: 10.3390/cancers17050907, PMID: 40075754 PMC11899122

[22] Kastritis E Leblond V Dimopoulos MA Kimby E Staber P Kersten MJ . Waldenström’s macroglobulinaemia: ESMO Clinical Practice Guidelines for diagnosis, treatment and follow-up. Ann Oncol. (2018) 29:iv270. doi: 10.1093/annonc/mdy322, PMID: 30285219

[23] Tam CS Opat S D’Sa S Jurczak W Lee HP Cull G . A randomized phase 3 trial of zanubrutinib vs ibrutinib in symptomatic Waldenström macroglobulinemia: the ASPEN study. Blood. (2020) 136:2038–50. doi: 10.1182/blood.2020006844, PMID: 32731259 PMC7596850

[24] Dimopoulos MA Opat S D’Sa S Jurczak W Lee HP Cull G . Zanubrutinib versus ibrutinib in symptomatic waldenström macroglobulinemia: final analysis from the randomized phase III ASPEN study. J Clin Oncol. (2023) 41:5099–106. doi: 10.1200/jco.22.02830, PMID: 37478390 PMC10666987

[25] Dimopoulos MA Tedeschi A Trotman J García-Sanz R Macdonald D Leblond V . Phase 3 trial of ibrutinib plus rituximab in Waldenström’s Macroglobulinemia. N Engl J Med. (2018) 378:2399–410. doi: 10.1056/NEJMoa1802917, PMID: 29856685

