# Supplementary material for: A real-world 10-year follow-up analysis of survival and safety of CD20-targeted therapy in patients with Waldenstrom macroglobulinemia
Source: Front Oncol. 2026 Mar 2;16:1733209. doi: 10.3389/fonc.2026.1733209 (PMC12989397; doi:10.3389/fonc.2026.1733209)
Supplement: Supplementary file 1 [file Table1.docx]

**Supplementary Table S1. Detailed baseline laboratory parameters by treatment group**

| Laboratory parameter | FCR group (n=50) | BR group (n=58) | R-CHOP group (n=12) | RTX group (n=8) | χ²/F-value | P-value |
| --- | --- | --- | --- | --- | --- | --- |
| Hemoglobin (g/dL, mean ± SD) | 10.73 ± 1.86 | 10.33 ± 2.15 | 10.25 ± 1.91 | 10.56 ± 1.84 | 0.424 | 0.736 |
| White blood cell count (×10⁹/L, mean ± SD) | 6.75 ± 2.26 | 6.92 ± 2.65 | 6.52 ± 2.38 | 6.85 ± 2.53 | 0.104 | 0.958 |
| Platelet count (×10⁹/L, mean ± SD) | 155.06 ± 48.57 | 148.86 ± 52.36 | 149.56 ± 53.24 | 147.75 ± 48.54 | 0.154 | 0.927 |
| Serum IgM (g/dL, mean ± SD) | 4.86 ± 2.14 | 6.53 ± 3.54 | 12.04 ± 4.11 | 5.17 ± 2.26 | 18.251 | <0.001 |
| Lactate dehydrogenase (U/L, mean ± SD) | 200.36 ± 75.56 | 214.86 ± 85.47 | 220.69 ± 90.24 | 205.89 ± 78.85 | 0.370 | 0.774 |
| β2-microglobulin (mg/L, mean ± SD) | 3.05 ± 1.07 | 3.36 ± 1.28 | 3.65 ± 1.44 | 3.17 ± 1.05 | 1.072 | 0.363 |
| Bone marrow infiltration (%, mean ± SD) | 18.45 ± 10.36 | 30.24 ± 15.77 | 45.18 ± 12.65 | 20.26 ± 10.31 | 16.107 | <0.001 |
